# Cryopreserved nanostructured fibrin-agarose hydrogels are efficient and safe hemostatic agents

**DOI:** 10.1038/s41598-024-70456-w

**Published:** 2024-08-21

**Authors:** Carlos Casado, Carmen Cepeda-Franco, Sheila Pereira Arenas, Maria Dolores Suarez, Miguel Ángel Gómez-Bravo, Miguel Alaminos, Jesús Chato-Astrain, Beatriz Fernández-Muñoz, Rafael Campos-Cuerva

**Affiliations:** 1 Unidad de Producción y Reprogramación Celular, Red Andaluza de Diseño y traslación de Terapias Avanzadas-RAdytTA, Fundación Pública Andaluza Progreso y Salud (FPS), Av. Américo Vespucio 15, 41092 Seville, Spain; 2 Instituto de Biomedicina de Sevilla/Hospital Universitario Virgen del Rocío/CSIC/Universidad de Sevilla, Seville, Spain; 3 Transplantation and Hepatobiliary Surgery Unit, Hospital Universitario Virgen del Rocío, Seville, Spain; 4 Servicio de Anatomía Patológica, Hospital Universitario Virgen del Rocío, Seville, Spain; 5 Tissue Engineering Group, Facultad de Medicina Universidad de Granada, Granada, Spain; 6 Instituto de Investigación Biosanitaria Ibs. Granada, Granada, Spain; 7 Departamento de Farmacia y Tecnología Farmacéutica, Facultad de Farmacia, Universidad de Sevilla, Seville, Spain; 8 Centro de Transfusiones, Tejidos y Células de Sevilla, Seville, Spain

**Keywords:** Cryopreservation, Hemostatic patch, Fibrin hydrogel, Liver resection, Trehalose, TachoSil, Liver, Surgery

## Abstract

Uncontrolled bleeding during surgery is associated with high mortality and prolonged hospital stay, necessitating the use of hemostatic agents. Fibrin sealant patches offer an efficient solution to achieve hemostasis and improve patient outcomes in liver resection surgery. We have previously demonstrated the efficacy of a nanostructured fibrin-agarose hydrogel (NFAH). However, for the widespread distribution and commercialization of the product, it is necessary to develop an optimal preservation method that allows for prolonged stability and facilitates storage and distribution. We investigated cryopreservation as a potential method for preserving NFAH using trehalose. Structural changes in cryopreserved NFAH (Cryo-NFAH) were investigated and comparative in vitro and in vivo efficacy and safety studies were performed with freshly prepared NFAH. We also examined the long-term safety of Cryo-NFAH versus TachoSil in a rat partial hepatectomy model, including time to hemostasis, intra-abdominal adhesion, hepatic hematoma, inflammatory factors, histopathological variables, temperature and body weight, hemocompatibility and cytotoxicity. Structural analyses demonstrated that Cryo-NFAH retained most of its macro- and microscopic properties after cryopreservation. Likewise, hemostatic efficacy assays showed no significant differences with fresh NFAH. Safety evaluations indicated that Cryo-NFAH had a similar overall profile to TachoSil up to 40 days post-surgery in rats. In addition, Cryo-NFAH demonstrated superior hemostatic efficacy compared with TachoSil while also demonstrating lower levels of erythrolysis and cytotoxicity than both TachoSil and other commercially available hemostatic agents. These results indicate that Cryo-NFAH is highly effective hemostatic patch with a favorable safety and tolerability profile, supporting its potential for clinical use.

## Introduction

Uncontrolled bleeding during surgery remains a major concern, as it can lead to high rates of mortality, morbidity and prolonged hospital stays^1^. Several blood control techniques have been developed in an effort to minimize the impact of blood loss during liver resection surgery; however, despite advances in surgical techniques, the reduction in bleeding during surgery is often transient and incomplete^2,3^. For this reason, the administration of hemostatic agents and other drugs is necessary to facilitate, enhance, and supplement or mimic the natural hemostasis mechanisms^4^. A hemostatic agent is a substance used to achieve hemostasis by accelerating the blood clotting process. To be suitable for surgical use, a hemostatic agent must meet specific requirements, including safety, effectiveness, applicability, low cost and regulatory approval^5^. Unfortunately, there is currently no single ideal product, making it necessary to develop new materials and technologies to effectively control and stop bleeding^6^.

The development of new products is motivated by the need for less invasive medical procedures, the goal of reducing the frequency of transfusions to minimize associated health complications, and the aim of improving cost-effectiveness across multiple surgical disciplines^6,7^. Fibrin sealant patches are the most efficient hemostatic agents for reducing the time to achieve hemostasis during liver resection^2^. This significantly contributes to improving patient stabilization and survival rates^8^.

Our previous work evaluated the efficacy of a novel hemostatic agent made from a nanostructured fibrin and type VII agarose hydrogel (NFAH)^9^. NFAH has previously been used as a scaffold biomaterial in several preclinical^10–12^ and clinical^13,14^ studies. This fibrin and agarose matrix is mainly derived from human plasma, has high flexibility, elasticity and mechanical strength^15,16^ and has been proven to be biodegradable and biocompatible^11,17^. However, in order to extend the half-life of this hemostatic agent, it is necessary to investigate a preservation method that maintains its structure and ensures long-term biological and therapeutic suitability.

The aim of the present study was to investigate a preservation method that would allow for longer stability and easier storage and distribution of NFAH for future commercialization. We also conducted in vitro and in vivo studies to further evaluate the efficacy and safety of cryopreserved NFAH (Cryo-NFAH). We first investigated whether cryopreservation would cause significant structural changes to the fibrin and agarose matrix of Cryo-NFAH that could affect its hemostatic efficacy. We then compared its in vitro safety profile with that of commercially available hemostatic agents: TachoSil^®^ (fibrinogen/thrombin-coated collagen patch), Hemopatch™ (polyethylene glycol‐coated collagen pad) and Surgicel^®^ (oxidized cellulose patch). Finally, we conducted an in vivo safety study of Cryo-NFAH versus TachoSil^®^ in a rat partial hepatectomy model.

## Material and methods

### Generation of the NFAH patch

The preparation of the NFAH was modified from our previously described method^9^. To generate the fibrin-agarose hydrogel (FAH), a 30 ml mixture was prepared as follows: 27 ml of human plasma and 0.5 ml of tranexamic acid (Amchafibrin 500 mg, Rottapharm, Milan, Italy) were added to a 50-ml conical tube. A solution containing 0.6 ml of 10% calcium chloride (B. Braun, Melsungen, Germany), 0.4 ml DPBS (Merck KGaA, Darmstadt, Germany) and 1.5 ml of melted 2% type VII agarose (Merck KGaA) was then added. After careful mixing, 5 ml of this solution was added to each well of a 6-well plate (Corning, New York, NY) and the plate was incubated at 37 °C for 2 h to allow gelation. Upon completion of this process, the hydrogels were covered with DPBS and maintained in the incubator at 37 °C for 24 h prior to nanostructuring. The Centro de Transfusiones, Tejidos y Celulas of Seville (CTTS, Sevilla, Spain), provided human plasma. The Andalusian Coordinator Ethical Committee for Biomedical Research through the Andalusian Public Health System Biobank approved their procurement (application code 32190054PV01) and all donors signed an informed written consent according to Spanish law (RD 1088/2005).

Nanostructuration is a mechanical biofabrication process in which hydrogels are subjected to compression and dehydration^15^. The fibrin-agarose hydrogel was placed between two 10-µm nylon mesh filters (NY-1009000; Merck KGaA) and compressed with extra thick western blotting filter paper (88620; Thermo Fisher Scientific, Waltham, MA) for at least 1 min and 40 s under a 250 g square glass. The final patch was then cryopreserved as described below or maintained in Ringer’s lactate solution (Fresenius Kabi AG, Bad Homburg, Germany) at room temperature until use.

### Cryopreservation

Manufactured NFAHs were immersed in 0.4 M trehalose solution for 2 h at room temperature. Subsequently, the NFAHs were stored at − 20 °C for 3 months. Cryopreserved NFAH (Cryo-NFAHs) were then thawed at room temperature at the time of use.

### Microscopy analysis

Structural analysis of the NFAHs was performed using light microscopy and scanning electron microscopy (SEM). Samples were fixed in formaldehyde for 24 h, dehydrated and embedded in paraffin. Subsequently, 4-µm-thick sections were cut on a microtome, deparaffinized in xylene, cleared in ethanol and stained using routine hematoxylin–eosin staining protocols (GHS316 and HT110116, Merck KGaA). Histological images were obtained, and the porosity of the NFAH was calculated using ImageJ software^18^. For SEM analysis, NFAH samples were fixed in 2.5% glutaraldehyde in cacodylate buffer followed by dehydration in increasing concentrations of acetone. After drying with liquid CO_2_ at high pressure, samples were sputter-coated with gold–palladium and analyzed using a Quanta 200 microscope in high vacuum mode (FEI, Eindhoven, The Netherlands). SEM images were recorded at magnifications of 1600×, 6000×, and 12,000×.

For histopathological analysis of hepatic sections, the samples were fixed in paraformaldehyde, embedded in paraffin and cut at a thickness of 2.5–4 µm. Sections were stained using hematoxylin–eosin and the Trichrome Stain (Masson) Kit (HT15-1KT, Merck KGaA) following standard methods.

The following histological variables were assessed by light microscopy: hemorrhage, inflammation (acute and chronic), necrosis, foreign body reaction, fibrosis, re-epithelialization, fibrin and mesothelial membrane. The categorization and scoring of liver injury were performed according to the criteria shown in Supplementary Table S1.

### Cytotoxicity

For the cytotoxicity assay, we adapted a previously described protocol^19^ (Supplementary Figure S1A). Briefly, the hemostatic agents Cryo-NFAH, TachoSil^®^ (Corza Medical, Linz, Austria), Hemopatch™ (Baxter, Deerfield, IL) and Surgicel^®^ (Ethicon, Johnson and Johnson MedTech, Summerville, NJ) were immersed in MSC medium consisting of DMEM low glucose (Merck KGaA) supplemented with 15% (v/v) FBS (Merck KGaA), 2 mM ultraglutamine (Lonza, Basel, Switzerland), 2.5 μg/ml amphotericin B (Merck KGaA) and 50 μg/ml gentamicin (Normon Laboratories, Madrid, Spain). After incubation at 37 °C for 24 h, the leachate was filtered through a 100-µm filter and a 0.22-µm filter, sequentially, to remove possible traces of the hemostatic agents. Human umbilical cord-derived mesenchymal stem cells (UC-MSCs) obtained from the Andalusian Public Health System Biobank were seeded at a concentration of 3000 cells/cm^2^ and incubated at 37 °C and 5% CO_2_ in MSC medium. After 24 h, the medium was replaced with the medium in which the hemostatic agents had previously been incubated. Cells cultured in MSC medium served as the control. The leaching medium was changed every two days. On days 1, 3 and 7, cell counts were performed in duplicate, and apoptosis and necrosis were assessed by flow cytometry using the Anexin V-FITC Kit (Miltenyi Biotec, Bergisch-Gladbach, Germany). Briefly, UC-MSCs were incubated with Anexin V-FITC and propidium iodide as indicated by the manufacturer. Sample acquisition was performed on the MACSQuant Analyzer 10 flow cytometer and the results were analyzed with MACS Quantify 2.10 software (both from Miltenyi Biotec). Events (> 10,000 single cells) were analyzed. The experiment was performed in triplicate using three independent UC-MSC samples. The use of human UC-MSCs was approved by the Andalusian Biomedical Research ethical committee, in compliance with current ethical guidelines and regulations (application code S2300454 and PEIBA internal code: 2026-N-23).

### Hemocompatibility

An adaptation of a previously developed protocol^20^ was used to assess hemolytic effects (Supplementary Figure S1B). First, human erythrocytes were isolated by centrifugation of whole blood obtained from healthy donors at 840 g for 20 min. After removing the plasma and buffy coat, a 1:25 dilution of erythrocyte concentrate was prepared in DPBS and 0.5 ml of this diluted erythrocyte solution was added to a 1.5-ml Eppendorf tube with a 0.25 cm^2^ square piece of hemostatic agent and incubated at 37 °C for 1 h in an orbital shaker at 50 rpm. The hemostatic agents were then centrifuged at 800 g for 15 min and the supernatants were collected. Finally, the free hemoglobin in supernatants was quantified using the Plasma/Low Hb System photometer (Hemocue AB, Ängelholm, Sweden). The hemostatic agents tested in this assay were Cryo-NFAH, TachoSil^®^, Hemopatch^®^ and Surgicel^®^. Each sample was analyzed in triplicate. Erythrocytes lysed with distilled water and unlysed erythrocytes in DPBS were used as positive and negative controls, respectively. The utilization of blood specimens was approved by the Andalusian Biomedical Research ethical committee, in compliance with established ethical guidelines and regulations (application code S2100047 and PEIBA internal code: 0405-N-21). Fresh, non-coagulated blood from healthy donors with citrate–phosphate-dextrose (CPD) as an anticoagulant was provided by the Transfusion, Tissue, and Cell Center of Seville. All donors signed an informed written consent.

### In vitro coagulation test

The in vitro coagulation test was conducted as reported^19^. Three batches of NFAH and Cryo-NFAH were manufactured, and three samples were analyzed from each batch. Specifically, a 0.5 × 0.5 cm square piece of each hemostatic agent (NFAH and Cryo-NFAH) was placed in a 1.5-ml Eppendorf tube (Supplementary Figure S2). Pre-warmed human blood was supplemented with CaCl_2_ (B. Braun Medical S.A., Madrid, Spain) at a final concentration of 20 mM and homogenized by inversion. Immediately, 1 ml of this mixture was rapidly added to each tube and placed in a water bath at 37 °C. The tubes were inverted every 30 s to check for coagulation. The coagulation time was established when the generated clot did not dislodge under the action of gravity for at least five seconds. A tube without hemostatic agent was included as a control for this test.

### Animal protocol and hepatic resection

Liver resection in rats was performed as described^9^. Wistar rats (*Rattus norvegicus*) were anesthetized (80 mg/kg ketamine and 10 mg/kg xylazine) by subcutaneous injection and maintained with isoflurane inhalation. The liver was exposed by a longitudinal laparotomy and the median hepatic lobe was resected through a 1.5-cm incision. Circular hemostatic agents 24 mm in diameter were then applied and the time to hemostasis was assessed. Time to haemostasis was defined as the time from application to cessation of blood extravasation through the resection surface.

Rats were housed in individually ventilated cages with free access to food and water, and analgesia was administrated as required. Body temperature and weight were measured and a protocol for monitoring signs of pain according to Morton and Griffiths criteria^21^. Animals were euthanized by cardiac puncture under deep anesthesia and assessed for post-operative bleeding, incidence of hematoma, hemostatic patch migration and intra-abdominal unwanted adhesions. Blood samples were taken for measurement of inflammatory factors by enzyme-linked immunosorbent assay (ELISA). Intra-abdominal adhesions were scored as follows: 0, no adhesion; 1, thin adhesions separable by gravity; and 2, thick adhesions not separable by gravity. Sections of injured liver tissue attached to the hemostatic agent were collected for histopathological analysis.

A total of 17 male Wistar rats (200–250 g) were used in the Cryo-NFAH versus NFAH efficacy study and 30 male rats were used in the Cryo-NFAH versus TachoSil^®^ safety study. The rats used in this study were obtained from Instituto de Biomedicina de Sevilla, located in Seville, Spain. All animals were housed and maintained in compliance with the Directive 2010/63/EU and Royal Decree 53/2013 laying down basic rules for the care and handling of research animals^22^.

### Approval for animal experiments

All institutional and national guidelines for the care and use of laboratory animals were followed. Animal care and experimental procedures were conducted according to “Guide for the Care and Use of Laboratory Animals” published by the Ministry of Agriculture, Fisheries and Food (R.D. 53/2013, Law 32/2007) and European Communities Council Directive 2010/63/EU. The protocol was approved by the Research Ethics Committee of University Hospital Virgen Macarena and Virgen del Rocio (internal reference: 1131‐N‐15). All authors complied with the ARRIVE guidelines.

### ELISA

Blood samples were collected in EDTA tubes (BD Bioscience, Franklin Lakes, NJ), centrifuged at 1480 g for 5 min and then stored at -80 °C in small aliquots to avoid repeated freeze–thaw cycles. Cytokine analysis was performed using the following commercially available ELISA kits: rat C-reactive protein (CRP) ELISA Kit (Catalog No. ELR-CRP, RayBiotech Inc., Norcross, GA), rat Interleukin 1 beta (IL-1β) ELISA Kit (Catalog No. E-EL-R0012, Elabscience, Houston, TX), and rat tumor necrosis factor alpha (TNF-α) ELISA Kit (Catalog No. CSB-E11987R, Cusabio Technology LLC, Houston, TX).

### Statistical analysis

Data are presented as mean ± SEM. Significance was determine using the Mann–Whitney U test or the Kruskal–Wallis analysis of variance test with Dunn’s post hoc multiple comparison tests. Categorical variables were analyzed using the Chi square test or Fisher’s exact test. The cytotoxicity assay’s statistical significance was assessed through a two-way ANOVA followed by Tukey’s multiple comparisons test. Significance variation in weight or temperature of animals after surgery was determined by a mixed effect model test. Differences were considered significant at *p* ≤ 0.05. All statistical analyses were performed using GraphPad Prism 9 (GraphPad Software Inc., San Diego, CA).

## Results

### Cryo-NFAH retains hemostatic efficacy

We have previously shown that freshly prepared NFAHs are potent hemostatic agents (Campos-Cuerva 2019). To facilitate large-scale production, storage and distribution, we evaluated different preservation methods. Trehalose has previously been proposed as an effective cryoprotectant for fibrin-agarose scaffolds at liquid nitrogen temperature^23^. Considering that not all surgical units and hospitals have easy access to a liquid nitrogen tank (− 196 °C), we decided to conduct a study using a conventional freezer for NFAH preservation at − 20 °C with trehalose. Efficient preservation of NFAH at this temperature would allow easier distribution and storage in hospitals that have conventional freezers at − 20 °C.

**Figure 1:**
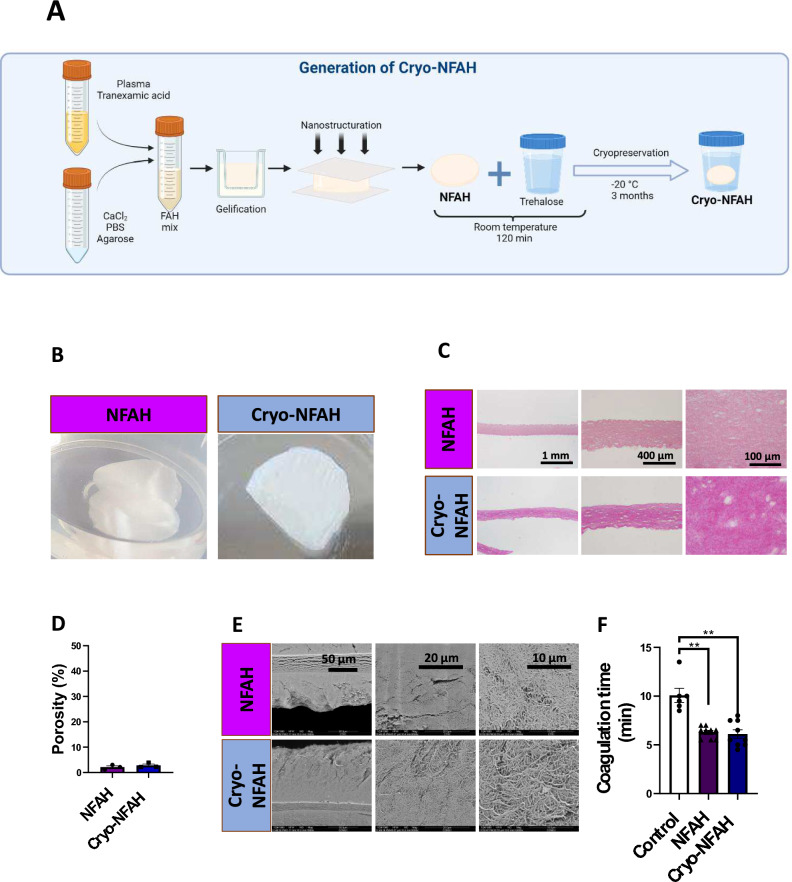
NFAH cryopreservation study. Samples were analyzed before (NFAH) and after (Cryo-NFAH) cryopreservation to check the integrity of the structure. (**A**) Explanatory scheme of the study. (**B**) Representative macroscopic images of samples. (**C**) Representative microscopic images of hematoxylin–eosin stained samples at ×40, ×100 and ×400 magnifications. (**D**) Porosity of the hydrogels. (**E**) Scanning electron microscopy analysis at ×1600; ×6000 and ×12,000 magnifications. (**F**) In vitro coagulation time assay.

Three different batches of NFAH were produced. For each batch, the NFAH samples were divided into two parts. One half was used for pre-cryopreservation histology by light and electron microscopy analysis, and the other half was cryopreserved at − 20 °C (Fig. 1A). The cryopreserved samples, termed Cryo-NFAH, were thawed after three months for post-cryopreservation histological analysis.

The results were similar for all batches analyzed. Macroscopic analysis revealed no obvious differences between cryopreserved and unpreserved NFAH, with apparently similar appearance and strength (Fig. 1B). Histological analysis showed that the structure of the Cryo-NFAH mesh was similar to that before cryopreservation. Hematoxylin–eosin analysis showed a Cryo-NFAH microscopic structure without large breaks, similar to the NFAH pre-cryopreserved control (Fig. 1C). There were no significant differences in the percentage of porosity before and after NFAH freezing (Fig. 1D and Supplementary Figure S3). Similarly, SEM analysis showed that Cryo-NFAH retained an extensive three-dimensional structure (Fig. 1E).

**Figure 2:**
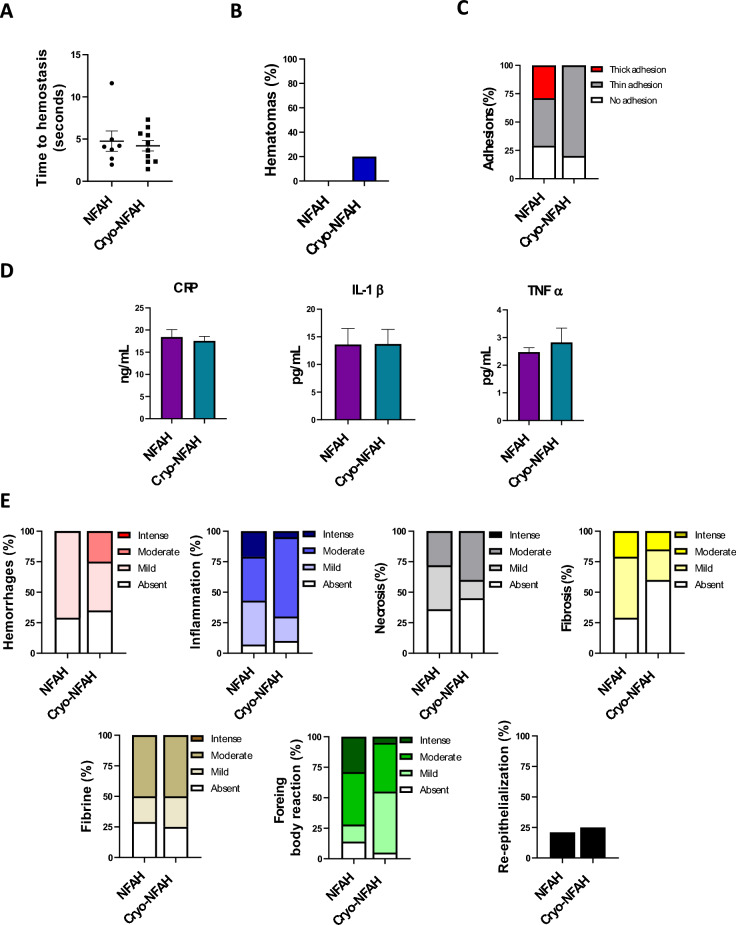
Efficacy and short-term safety study of Cryo-NFAH. (**A**) Time to reach hemostasis by application of hemostatic agents in a severe liver resection model (1.5 cm liver incision). Results are presented as ± SEM, Mann–Whitney U test (*p* > 0.99). (**B**) Percentage of rats with hepatic hematoma 24 h after treatment application. (**C**) Adhesion grade of hemostatic agents. Adhesion in each sample was scored from 0 to 2: 0, no adhesion; 1, thin adhesion separable by gravity; and 2, thick adhesion not separable by gravity. (**D**) Levels of inflammatory factors in blood from rats subjected to liver resection and treated with hemostatic agents at the moment of sacrifice (24 h): C-reactive protein (CRP), interleukin-1 beta (IL-1β) and tumor necrosis factor-alpha (TNF-α). Data represent mean ± SEM and were analyzed using (N = X–Y); *p < 0.05; **p < 0.01, ***p < 0.001. (**E**) Histopathological variables of animals treated with NFAH and Cryo-NFAH after partial hepatectomy.

To investigate whether cryopreservation affects the efficacy of NFAH, we performed hemostatic efficacy assays. We first tested Cryo-NFAH in an in vitro coagulation test, and found that the clotting time was significantly lower with Cryo-NFAH than with endogenous coagulation (*p* = 0.002). Notably, no differences were found between fresh NFAH and Cryo-NFAH treatments: mean clotting times were 6.11 ± 0.43 min and 6.27 ± 0.21 min for Cryo-NFAH and fresh NFAH, respectively, indicating that cryopreservation did not affect hemostatic efficacy (Fig. 1F). Additionally, the hemostatic performance of Cryo-NFAH was compared in a rat partial hepatectomy model. Animals underwent a 1.5-cm liver resection and hemostatic agents were applied. No significant differences were found between groups (*p* > 0.99): time to hemostasis was 4.75 ± 1.2 s (N = 7, range = 2–11.6) in the NFAH group and 4.21 ± 0.62 s (N = 10, range = 1.4–7.3) in the Cryo-NFAH group (Fig. 2A).

### Cryo-NFAH has a favorable in vivo safety profile

Having demonstrated that cryopreservation of NFAH with trehalose did not alter its hemostatic efficacy in vitro and in vivo, we evaluated the safety profile of Cryo-NFAH in rats. Twenty-four hours after partial hepatectomy, visual inspection of the wound revealed the presence of hematomas in 20% of rats treated with Cryo-NFAH (Fig. 2B). Evaluation of unwanted adhesions to surrounding healthy tissues showed no thick adhesions in Cryo-NFAH-treated animals, whereas 25% of adhesions were thick in animals treated with fresh NFAH (Fig. 2C). We next measured the levels of inflammatory factors in blood samples taken at sacrifice by ELISA. No significant differences were found between the NFAH and Cryo-NFAH groups for the measured cytokines (p^CRP^ = 0.64, p^IL-1β^ = 0.98, p^TNF‐α^ = 0.6) (Fig. 2D). Furthermore, both NFAH treatments (NFAH and Cryo-NFAH) showed overall similar histopathological results (Fig. 2E).

**Figure 3:**
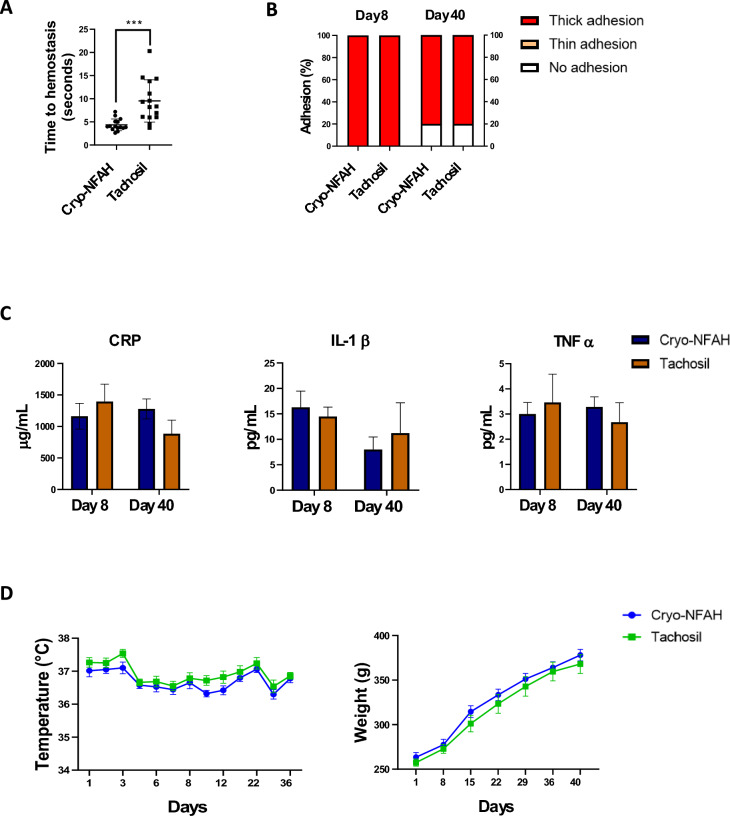
Long-term Cryo-NFAH safety study. (**A**) Time to reach hemostasis by application of hemostatic agents in the severe liver resection model (1.5 cm liver incision). Results are presented as ± SEM, Student’s t test (***p < 0.001). (**B**) Degree of adhesions caused by the application of the different hemostatic agents at days 8 and 40 post-surgery. The adhesion in each sample was classified with a value between 0 and 2: 0, without adhesions; 1, thin adhesions separable by gravity; and 2, thick adhesions not separable by gravity. (**C**) Levels of C-reactive protein (CRP), interleukin-1 beta (IL-1β) and tumor necrosis factor-alpha (TNF-α) in blood at the moment of sacrifice (8 and 40 days). (**D**) Evolution of the temperature and body weight of the animals throughout the study.

Having verified that the safety profile was similar between Cryo-NFAH and freshly prepared NFAH, we performed a long-term safety study (40 days after surgery) to compare Cryo-NFAH with the widely used fibrin-based commercial hemostatic agent TachoSil^®^. We first compared the hemostatic efficacy of Cryo-NFAH and TachoSil^®^ in the partial hepatectomy model, as before. The difference in time to hemostasis between groups was significant (*p* = 0.0002) with a superior hemostatic profile for Cryo-NFAH: mean time to hemostasis was 9.52 ± 1.17 s (N = 15, range = 3.74–20.3) in the TachoSil^®^ group and 4.36 ± 0.32 s (N = 15, range = 2.61–7.2) in the Cryo-NFAH group (Fig. 3A).

The wounds were then sutured and a follow-up analysis was performed to assess the potential occurrence of adverse effects. Animals were sacrificed at 8 and 40 days after treatment. With regards to hematomas, the analysis did not identify any hematoma at any time of sacrifice in any of the groups (data not shown), indicating that the hematomas occurring 24 h after Cryo-NFAH application resolve within the first week. However, there were postoperative adhesions on day 8 and day 40 post-surgery, with a similar profile between groups (Fig. 3B).

The inflammatory factors CRP, IL-1β and TNF-α were measured in blood at the time of sacrifice (Fig. 3C), which revealed no significant differences between groups at either time point (*p* ^8 days CRP^ = 0.71, *p* ^8 days IL-1β^ = 0.62, *p* ^8 days TNF‐α^ = 0.71, *p* ^40 days CRP^ = 0.37, *p* ^40 days IL-1β^ = 0.6, *p* ^40 days TNF‐α^ = 0.79). Overall, the histopathological analysis identified a lower degree of fibrosis and higher re-epithelialization and mesothelial membrane formation at day 40 in the Cryo-NFAH group, indicating greater efficacy in promoting healing and reducing scarring (Supplementary Figure S4 and S5). No necrosis or fibrin deposition was observed in any sample from animals treated with either hemostatic agents.

Body temperature and weight gain were also recorded (Fig. 3D). Postoperative body temperature of the animals remained within the normal range (36–38 °C) throughout the study in both groups. No significant differences in postoperative body temperature (*p* = 0.31) or weight gain (*p* = 0.9973) were observed between animals treated with Cryo-NFAH or TachoSil^®^. In addition, the animals gained body weight progressively throughout the study. No animal died or had any apparent adverse reaction during the study with any of the treatments.

### Hemo- and cytocompatibility in vitro studies with Cryo-NFAH

**Figure 4:**
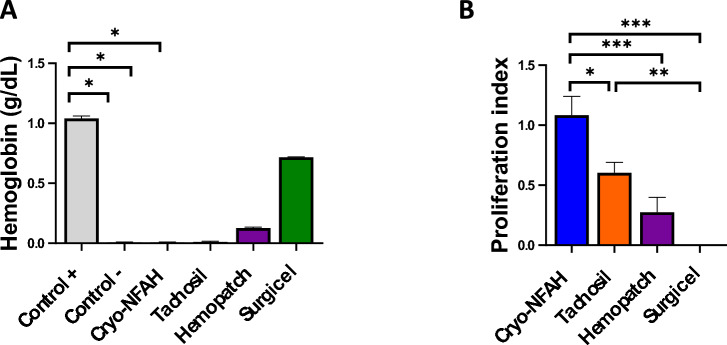
In vitro safety studies. (**A**) Hemoglobin released by erythrolysis caused by hemostatic agents. (**B**) Proliferation index of human umbilical cord-mesenchymal stem cells (UC-MSCs) in the presence of hemostatic agents. Data represent mean ± SEM. Kruskal–Wallis test with Dunn’s post hoc multiple comparison test and a two-way ANOVA followed by Tukey’s multiple comparisons test were performed for erythrolysis and proliferation index assays, respectively; *p < 0.05; **p < 0.01, ***p < 0.001.

Direct contact of hemostatic dressing with blood cells can have negative biological effects. For this reason, we assessed the hemolytic activity of the materials by measuring hemoglobin release from human erythrocytes upon in vitro contact with the hemostatic dressings. In contrast to Hemopatch^®^ and Surgicel^®^, analysis of the data showed that Cryo-NFAH did not cause erythrolysis, with free hemoglobin levels similar to the negative control (unlysed erythrocytes in DPBS) (Fig. 4A).

Finally, cytotoxicity tests were performed to determine whether Cryo-NFAH and other commercially available hemostatic agents could cause damage to human cells (loss of viability and/or variation in growth). Three UC-MSC lines from different donors were used for the analysis (Supplementary figure S6A). To compare the results obtained from the three cell lines and due to their different growth rates, we calculated a normalized indicator called proliferation index (PI) using the control group data for each cell line (PI = number of cells obtained within the hemostatic treatment/ number of cells obtained within the control group). Thus, a value less than 1 indicates that the hemostatic agent has a toxic effect on the cells, a value of 1 indicates no effect, and a value greater than 1 indicates a positive effect on cell proliferation. After 7 days, results showed that the cellular growth with the Cryo-NFAH leachate was similar to that of the control group. By contrast, TachoSil^®^ and Hemopatch^®^ leachates showed significant decreased proliferation (*p* = 0.01 and *p* < 0.0001, respectively) and Surgicell^®^ completely abolished it (Fig. 4B). Similarly, apoptosis and necrosis were lower in Cryo-NFAH than in the other commercial hemostatic agents, as measured by annexin V and propidium iodide staining (Supplementary Figure S6B).

## Discussion

Our previous research on the hemostatic efficacy of NFAH was performed with freshly prepared hydrogels^9^. However, since most of the NFAH content is human plasma, whose proteins and coagulation factors significantly decrease after 14 days of storage at 4 °C^24^, and that fibrin hydrogel scaffolds biodegrade in PBS after few weeks^25,26^, we do not expect a long shelf life for NFAH, either at room temperature or at 4 °C. Oppositely, cryopreservation and lyophilisation are commonly used to increase the shelf-life of human plasma-derived products^27^. Therefore, to overcome this limitation and make NFAH readily available for unplanned surgery, we investigated the use of a cryopreservation method. An important advantage of cryopreservation is that it can extend the shelf life of the product and ensure its immediate availability, as it can be stored in hospitals and point-of-care sites until needed.

Among the various cryoprotective agents used in tissue engineering, trehalose has been shown to be safe and well tolerated in humans^28^ with interesting bioprotective properties as a protein stabilizer capable of preventing protein aggregation^29^. Furthermore, this disaccharide has been reported as a suitable cryoprotectant for cryopreservation of fibrin-agarose hydrogels in liquid nitrogen^23^. Cryopreservation of NFAH in liquid nitrogen is an alternative to fresh preparation; however, liquid nitrogen storage systems are not common in hospitals and point-of-care sites. Our stability study of Cryo-NFAH at − 20 °C was designed to find a more accessible storage condition. Moreover, this temperature would facilitate transport logistics from the manufacturing center to the requesting hospital unit. Our analysis showed positive macro- and microscopic results, indicating that storage at − 20 °C is favorable, with no significant changes in the structure and porosity of the hydrogels after cryopreservation.

We performed hemostatic efficacy tests and a short-term safety study to compare Cryo-NFAH with freshly-prepared NFAH. No significant differences were found in the time to hemostasis, confirming the − 20 °C temperature as a favorable long-term storage condition. Hematoma formation was observed with the use of Cryo-NFAH at the time of sacrifice; however, the incidence rate was lower than that with TachoSil^®^ and Hemopatch^®^ in the partial hepatectomy model^9^. In addition, as shown in our long-term safety study, hematomas resolved 8 days after application. In contrast to fresh NFAH, rats treated with Cryo-NFAH did not have thick adhesions. A possible explanation for this finding could be the residual presence of trehalose in the Cryo-NFAH dressings, which has been shown to reduce post-operative adhesions^30^.

Similar results for histopathological variables were found in rats treated with NFAH and Cryo-NFAH hemostatic agents after partial hepatectomy. The Cryo-NFAH treatment group showed moderate hemorrhage consistent with the presence of hematoma. By contrast, a slightly more intense inflammatory and foreign body reaction response was seen in the fresh NFAH group, consistent with the higher percentage of thick adhesions found in this group. Overall, both NFAH groups showed better histopathological results than TachoSil^®^ and Hemopatch^®^ analyzed in our previous study^9^.

We confirmed the safety of Cryo-NFAH as a hemostatic patch in a long-term study compared with TachoSil^®^, a widely used hemostatic patch made of equine collagen coated with human fibrinogen and thrombin. TachoSil^®^ was chosen because it produces fibrin by fibrinogen conversion, is similar in composition to NFAH, and has one of the most favorable safety results among commercially available hemostatic agents^31,32^. The hemostatic efficacy of Cryo-NFAH versus TachoSil^®^ was also tested in the partial hepatectomy model in rats. The superior hemostatic effects shown by Cryo-NFAH are in line with our previous findings with fresh NFAH^9^.

The long-term safety study showed no significant differences between groups in the percentage of hematomas, adhesions and inflammatory factors. Histopathological analysis identified a lower degree of fibrosis and greater re-epithelialization and mesothelial membrane formation at day 40 in the Cryo-NFAH group, suggesting greater efficacy in promoting healing and reducing scarring.

Analysis of the potential cytotoxic effects of Cryo-NFAH on human cells revealed the striking result that cell growth with the Cryo-NFAH leachate was considerably higher than with other commercially available hemostatic leachates, with lower apoptotic and necrotic rates. It has been previously reported^33,34^ that fibrin has a favorable effect on cell proliferation, likely through the release of growth factors contained in the fibrin clot^35^, as well as through cell-receptor mediated interactions with fibrin^36,37^. Additionally, our hemocompatibility study revealed no hemolytic effect of Cryo-NFAH. The results from these in vitro tests indicate that Cryo-NFAH has less cytotoxic and hemolytic activity than other commercially available hemostatic patches and confirm the favorable safety profile of Cryo-NFAH.

Overall, this study showed a preservation method for NFAH that allows its distribution and storage at the point of care and has demonstrated that Cryo-NFAH is an effective and safe hemostatic patch for liver surgery. We believe that our results represent an important step towards the commercialization of NFAH as a promising hemostatic agent for liver resection and other surgical procedures. However, larger safety studies including both male and female individuals are desirable.

### Supplementary Information

Supplementary Figures.

Supplementary Table 1.

## Data Availability

The datasets used and/or analysed during the current study are available from the corresponding author on reasonable request.

## Abbreviations

NFAH: Nanostructured fibrin-agarose hydrogel
Cryo-NFAH: Cryopreserved nanostructured fibrin-agarose hydrogel
FAH: Fibrin and agarose hydrogels
DPBS: Dulbecco’s phosphate-buffered saline
SEM: Scanning electron microscopy
DMEM: Dulbecco’s Modified Eagle’s Medium
FBS: Fetal bovine serum
UC-MSCs: Mesenchymal stem cells from umbilical cord
CO_2_: Carbon dioxide
CaCl_2_: Calcium chloride
ELISA: Enzyme-linked immunosorbent assay
EDTA: Ethylenediaminetetraacetic acid
CRP: C reactive protein
IL-1β: Interleukin 1 beta
TNF-α: Tumor necrosis factor alpha
