# Supplementary material for: Cryopreserved nanostructured fibrin-agarose hydrogels are efficient and safe hemostatic agents
Source: Sci Rep. 2024 Aug 21;14:19411. doi: 10.1038/s41598-024-70456-w (PMC11339259; doi:10.1038/s41598-024-70456-w)
Supplement: Supplementary file 1 — Supplementary Figures. [file 41598_2024_70456_MOESM1_ESM.pdf]

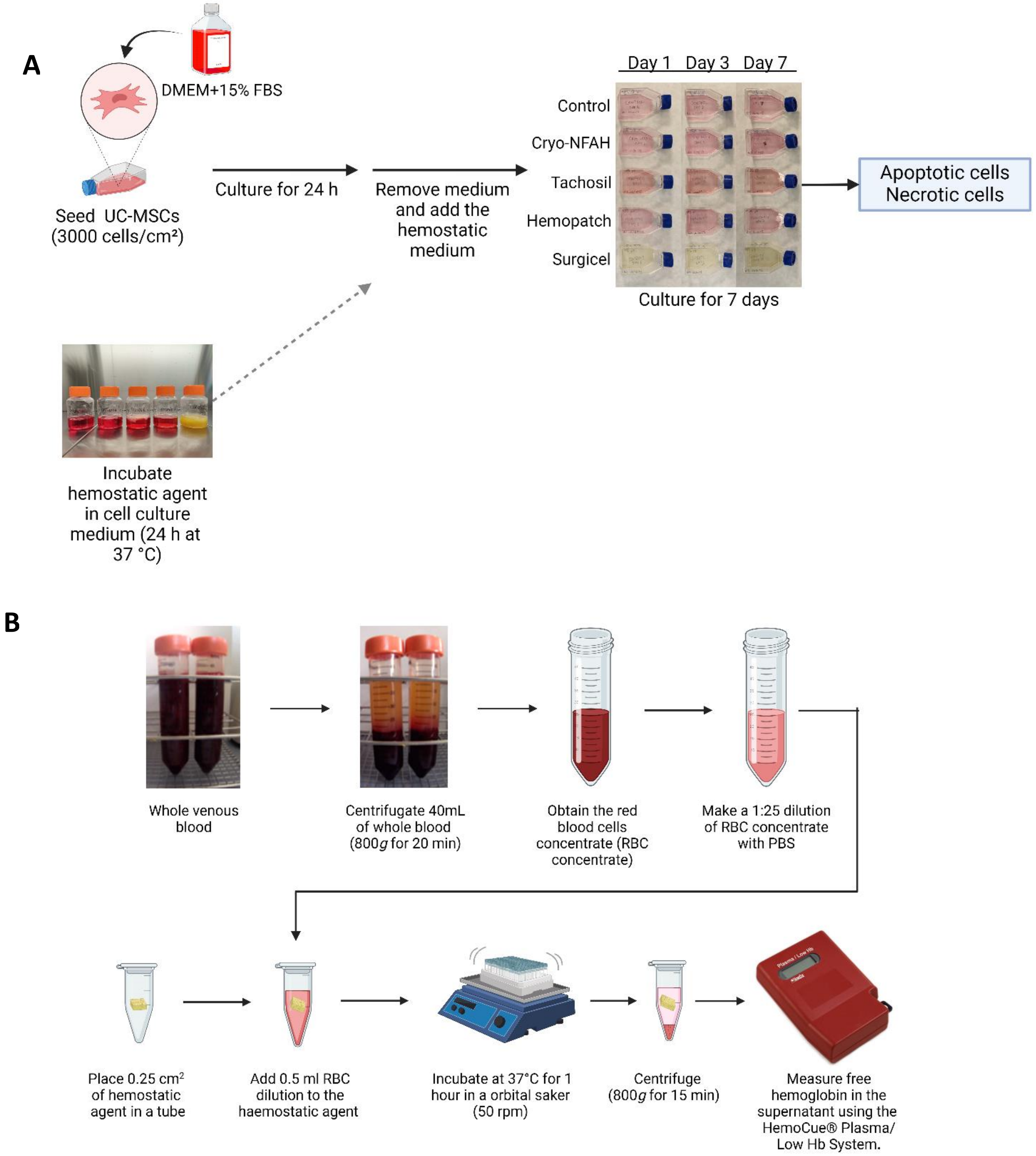

Figure S1. In vitro safety studies. A) Scheme of the cytotoxicity assay. The hemostatic agents were placed in culture medium at a concentration of 10 mg/ml and the umbilical cord-mesenchymal stem cells (UC-MSCs) were seeded at 3,000 cells/cm^2. After 24 hours in the incubator at 37ºC and 5% CO_2, the hemostatic agents were removed and the solutions were filtered and added to the cells. At days 1, 3 and 7, cells were harvested to count number of live cells and to analyze apoptotic and necrotic cells by flow cytometry using the Annexin V/propidium iodide kit. **B)** Protocol used for hemocompatibility test. The diagram shows the process followed to determine erythrolysis caused by the hemostatic agents. The Plasma/Low Hb System (Hemocue) was used for the quantitative determination of the released hemoglobin in the samples (n = 3). This image was created with BioRender (https://biorender.com/).

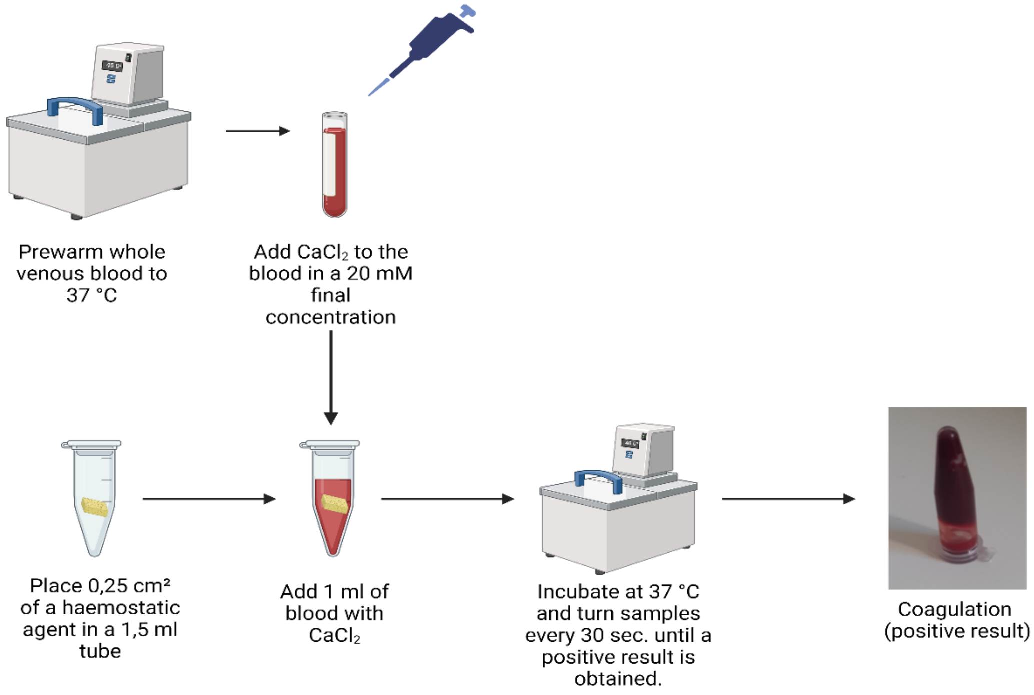

Figure S2. In vitro coagulation test. A 0.5 × 0.5cm square piece of a hemostatic agent was placed into a 1.5 ml Eppendorf tube. Subsequently, 1 ml of pre-warmed blood with 20 mM $CaCl_2$ was added to the tube. The tube was then placed in a water bath at 37°C. Coagulation time was established when the generated clot failed to dislodge due to the action of gravity. This image was created with BioRender (https://biorender.com/).

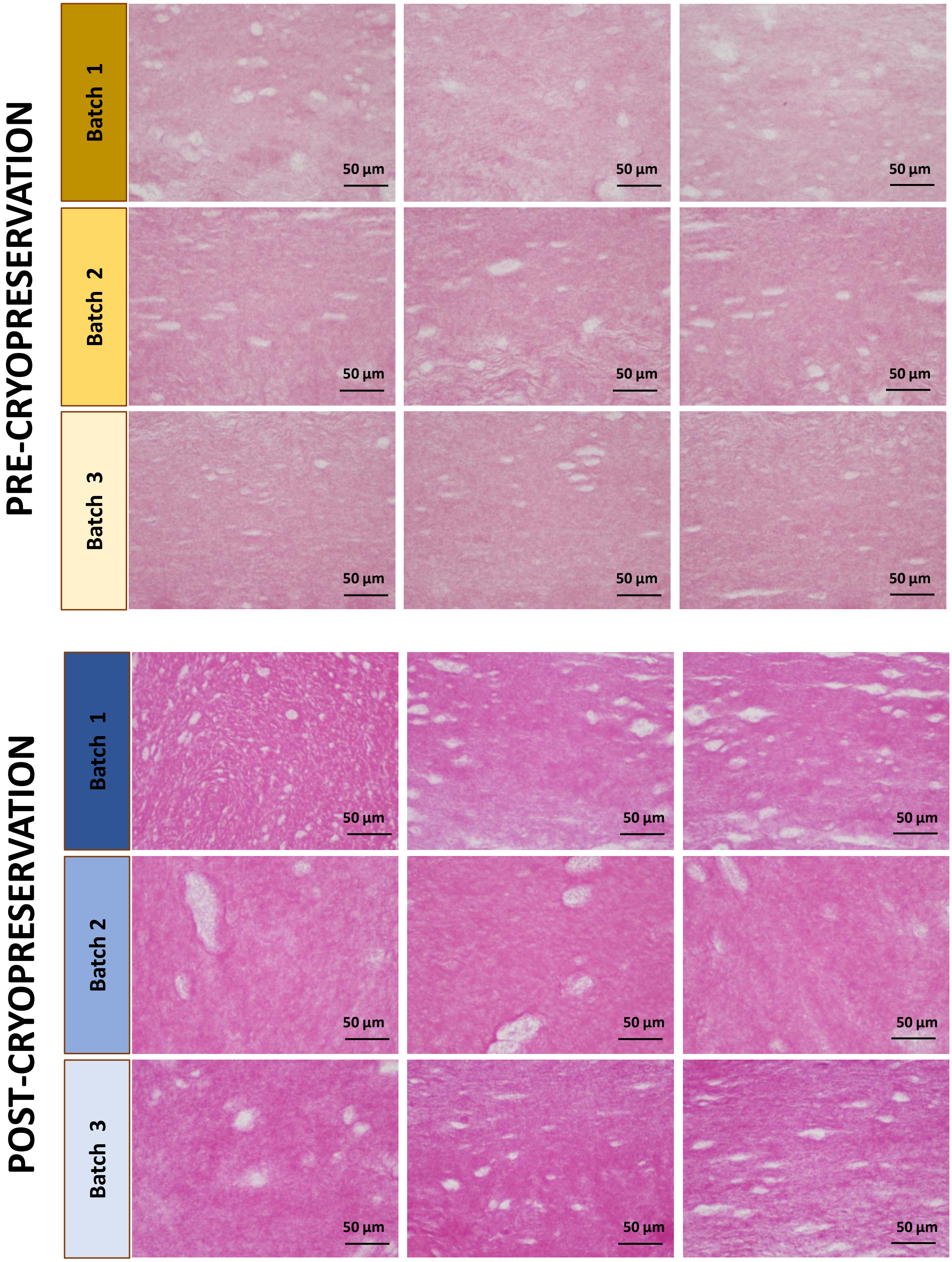

Figure S3. Representative images of porosity in NFAH. Three samples of different manufactured batches of NFAH were stained with hematoxylin-eosin and analyzed by light microscopy before (pre-cryopreservation) and after 3 month cryopreservation (post-cryopreservation). Scale bar: 50 µm

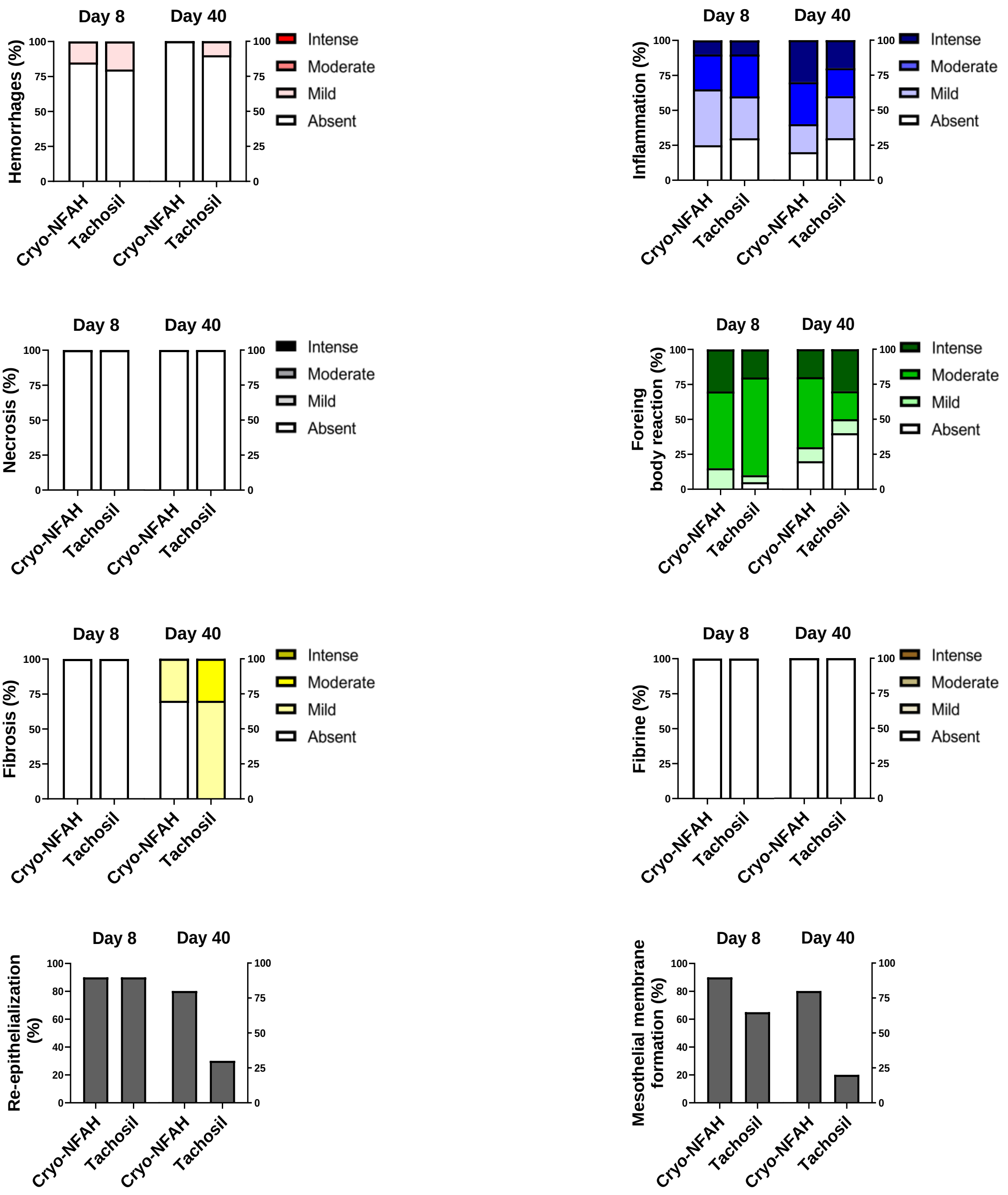

Figure S4. Histopathological analysis of hepatic parenchyma after treatments with hemostatic agents in rats with partial hepatectomy. Percentage of rats with hemorrhage, inflammation, necrosis, foreign body reaction, fibrosis, fibrin, re-epithelialization and mesothelial membrane formation after Cryo-NFAH and Tachosil treatments. Each sample was classified as absent, mild, moderate, or severe (see Supplementary Table 1 for the categorization in Materials and Methods).

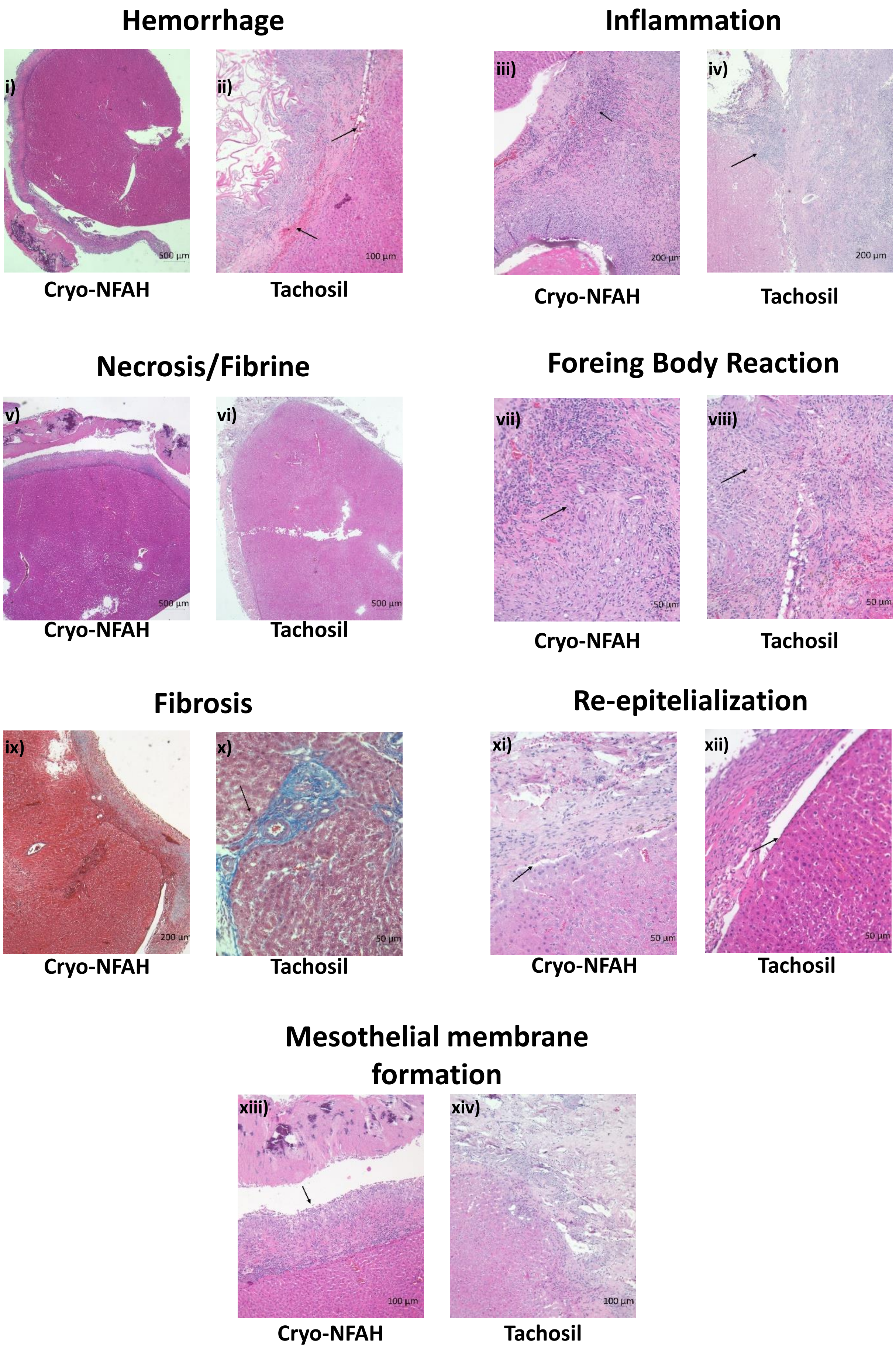

Figure S5. Histopathological analysis. Representative histological images at day 40 post-surgery showing: (i) absent and (ii) mild hemorrhage; (iii-iv) severe inflammation; (v-vi) absent necrosis and fibrine; (vii) moderate and (viii) severe foreign body reaction; (ix) absent and (x) moderate fibrosis; (xi) presence and (xii) absence of re-epithelialization; and (xiii) presence and (xiv) absence of mesothelial membrane formation.

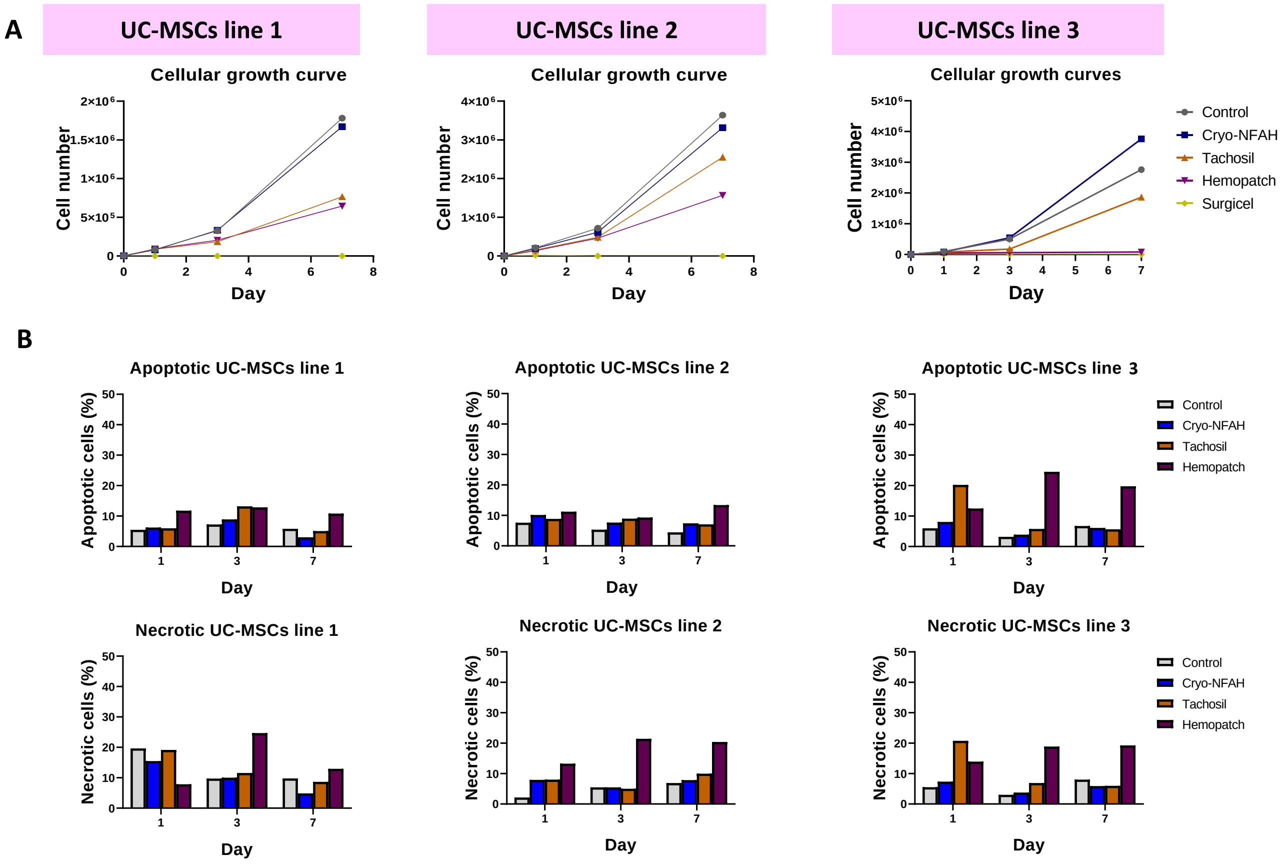

Figure S6. Cytotoxicity assay. **A)** Cell growth curves of human umbilical cord-mesenchymal stem cells (UC-MSCs) in the presence of hemostatic agents. **B)** Additional quantifications of the cytotoxic effect of hemostatic agents measured by Annexin V and propidium iodide staining. The percentages of apoptotic and necrotic cells are shown. It was not possible to perform flow cytometry on UC-MSCs incubated with Surgicel leaching solution due to the impossibility of recovering a sufficient number of cells.
