# Supplementary material for: Cryopreserved nanostructured fibrin-agarose hydrogels are efficient and safe hemostatic agents
Source: Sci Rep. 2024 Aug 21;14:19411. doi: 10.1038/s41598-024-70456-w (PMC11339259; doi:10.1038/s41598-024-70456-w)
Supplement: Supplementary file 2 — Supplementary Table 1. [file 41598_2024_70456_MOESM2_ESM.pdf]

TABLE S1. Histopathological criteria

Variable	Category (Scoring)			
	Absent (0)	Mild (1)	Moderate (2)	Intense (3)
**Hemorrhage**	Groups of erythrocytes that come together without forming a free zone of hemorrhage	Area of hemorrhage < 1 mm diameter	Area of hemorrhage 1–2.5 mm diameter and less than x40 hpf of depth	Area of hemorrhage >2.5 mm diameter or more than x40 hpf of depth
**Inflammation**	-	Few granulocytes	Some infiltrates, perivascular cuffing	Massive infiltration
**Necrosis**	-	0.1–0.4 hpf	0.5–1.2 hpf	>1.2 hpf
**Foreign Body Reaction (FBR)**	Foreign body granuloma formation consists of macrophages, multinucleated foreign body giant cells (macrophage fusion), fibroblasts, and angiogenesis.	Area of FBR > 1 mm diameter.	Area of FBR 1–2.5 mm diameter and less than x40 hpf of depth.	Area of FBR > 2.5 mm diameter or more than x 40 hpf of depth.
**Fibrosis**	-	0.1–0.4 hpf	0.5–1.2 hpf	>1.2 hpf
**Fibrin**	-	0.1–0.4 hpf	0.5–1.2 hpf	>1.2 hpf

Note. hpf: high-power fields.

Variable	Categorization	
**Re-epithelization**	Presence	Absent
**Mesothelial membrane formation (MMF)**	Presence	Absent
